# Stimuli-Responsive Nanocarriers as Next-Generation on-Demand Drug Delivery Systems for Cancer Therapy: Mechanistic Insights, Trigger Modalities, and Translational Challenges

**DOI:** 10.3390/pharmaceutics18070800

**Published:** 2026-06-29

**Authors:** Ahmed Abdulkarim Y. Alaysereen, Moath Mahmoud E. Daoud, Maha Munawar Alhoda M. Bader Alhoda, Ali Husain Ali Zayer, G. Roshan Deen

**Affiliations:** Materials for Medicine Research Group, School of Medicine, Royal College of Surgeons in Ireland, Medical University of Bahrain (RCSI-Bahrain), Busaiteen P.O. Box 15503, Bahrain; 22202180@rcsi-mub.com (A.A.Y.A.); 22202434@rcsi-mub.com (M.M.E.D.); 23201085@rcsi-mub.com (M.M.A.M.B.A.);

**Keywords:** stimuli-responsive polymers, tumor microenvironment, pH-responsive, redox-responsive, enzyme-responsive, on-demand drug delivery, cancer nanomedicine

## Abstract

Chemotherapy has been used in cancer treatment for decades; however, standard chemotherapy treatments still have significant weaknesses, including collateral damage to healthy tissue, rapid development of drug resistance, and dose-limiting toxicity that limits therapeutic value. There is now an alternative approach using polymer materials that are responsive to biological stimuli that will allow for improved treatment of cancer while avoiding the limitations. Responsive polymer materials are designed to be inert during circulation until they reach their site of action; then, they will respond to specific triggers. These smart carriers respond to stimuli present in the tumor microenvironment (e.g., low pH, high glutathione levels, and increased proteolytic activity) or external stimuli applied at the bedside (e.g., localized heat, light, ultrasound, and applied magnetic fields). In both cases, there is a consistent pattern where the drug is released exactly where/when it is needed, with minimal drug release occurring outside that location and timeframe. Therefore, it is theorized that the use of polymeric-based delivery systems with stimuli-regulated drug release will significantly increase the concentration of drug delivered intratumorally, decrease the drug toxicity, and provide a potential mechanism to overcome the development of multidrug resistance from a variety of cancer treatments. To date, various types of responsive polymers have been developed and could be combined to give rise to a wide variety of different vehicle systems (e.g., micelles, nanogels, hydrogels, and hybrid delivery systems), with many of these carriers designed to respond to multiple stimuli simultaneously. Nonetheless, significant challenges remain in the clinical application of these materials due to tumor heterogeneity, immune system interactions, reproducibility issues, polymer chemistry advances, surface chemistry, and other interaction mechanisms. As a result of all of these evolving regulatory systems, as well as some of the emerging areas of polymer chemistry and surface engineering, theranostic integration will allow for new routes to provide therapy for patients with cancer. Additionally, because of these scientific advances, there will also be more opportunities to provide targeted, controllable, and on-demand treatments to patients using stimuli-responsive polymers.

## 1. Introduction

Every year, cancer still kills millions of people. Even though surgery, radiation therapy, chemotherapy, and newer immunotherapies are getting better all the time, some problems still exist. Drugs are still blunt tools that do not work well because they do not target specific cells, damage healthy tissue, and cause resistance, which makes them less effective [1]. In short, therapeutic agents often spread to healthy organs just as easily as they do to tumors, which makes the therapeutic window smaller and the toxicity higher [2].

Researchers have turned to stimuli-responsive polymers to fix these problems. These adaptive systems can “read” their surroundings and respond in a manner that differs from that of regular carriers. Acidity, excessive glutathione, or enzymes that work too hard are examples of internal triggers that can be utilized in the tumor microenvironment. Light, heat, and ultrasound from outside sources can also be used to control when and where drugs are released. This means that therapy can only be initiated where the disease is present [3].

Currently, there are several different ways to design carriers. Amphiphilic copolymers form micelles that remain together in the blood but break apart when specific triggers occur, releasing their contents on demand [4]. More advanced nanocarriers utilize both pH-responsive and redox-cleavable components to prevent early release and enhance the accuracy of the process [5]. Biopolymer-based hydrogels offer an alternative approach; when implanted locally, they can either swell or break down in response to physiological changes, serving as adaptable “depots” for prolonged delivery [6]. However, implementing this in the clinic remains challenging. Tumor heterogeneity, the stability of treatments in vivo, immune interactions, and reproducibility among manufacturing batches present further challenges [7].

This review identifies the mechanisms that govern stimuli-responsive polymeric nanocarriers, both intrinsic and extrinsic triggering strategies, and their applicability in targeted treatment of cancer and assesses the translational challenges.

## 2. Mechanisms of Drug Release and Targeting

Targeted drug delivery and the specificity of drug accumulation in the body are broad concepts that can be achieved through various systems, each with their own set of mechanisms. These mechanisms enable the selection of the most appropriate delivery method based on the type of disease or pathology being treated [8]. There are various mechanisms for targeted drug release and delivery in cancer, encompassing different strategies to enhance therapeutic precision and efficacy, which are described below.

### 2.1. Passive Targeting via the Enhanced Permeability and Retention (EPR) Effect

Over the last 30 years, the rise of cancer nanomedicines has resulted in hundreds of papers and numerous authorized therapies for solid and blood malignancies, with the enhanced permeability and retention (EPR) effect being critical for targeted drug delivery in solid tumors [9]. The EPR concept emphasizes that solid tumors often induce angiogenesis, leading to blood vessels with defective endothelium and increased permeability. Furthermore, factors such as bradykinin, nitric oxide/peroxynitrite, prostaglandins, and vascular permeability factors, among many others, promote macromolecule extravasation and increase permeability into tumor tissues, resulting in passive diffusion of the drug to the tumor [10]. Furthermore, obstructed lymphatic arteries and reduced lymphatic clearance cause macromolecular medicines to remain in tumor tissues for extended periods, resulting in greater drug bioavailability at the tumor site [11].

Nano-sized medicines must be precisely developed for successful cancer therapy, with a focus on size and surface properties. Agents should ideally be less than 300 nm to avoid rapid clearance by the liver and reticuloendothelial system (RES); renal excretion also has to be taken into account, as agents larger than 6 nm are poorly filtered by the kidneys. Maintaining a nearly neutral charge and using hydrophilic coatings, such as polyethylene glycol, is critical for increasing circulation time. The ideal size range of 10 nm to 200 nm enables deeper tissue penetration and the delivery of appropriate therapeutic payloads [12]. This allows for maximal accumulation in the tumor. The concentration of the medicine in the tumor compared to the blood (T/B ratio) can be 10 to 30 times greater. When delivered through a tumor-feeding artery, the T/B ratio for styrene-maleic acid copolymer-conjugated neocarzinostatin (SMANCS)/Lipiodol can increase by up to 2000 times, allowing for precision targeting [13]. Pharmacological drugs that increase bradykinin levels are employed to augment EPR effects, as well as physical approaches such as moderate heat and photodynamic therapy, which produce reactive oxygen species and lower tumor fluid pressure. These approaches collaborate to enhance the therapeutic effectiveness of nanomedicines through tumor targeting [14].

**Figure 1:**
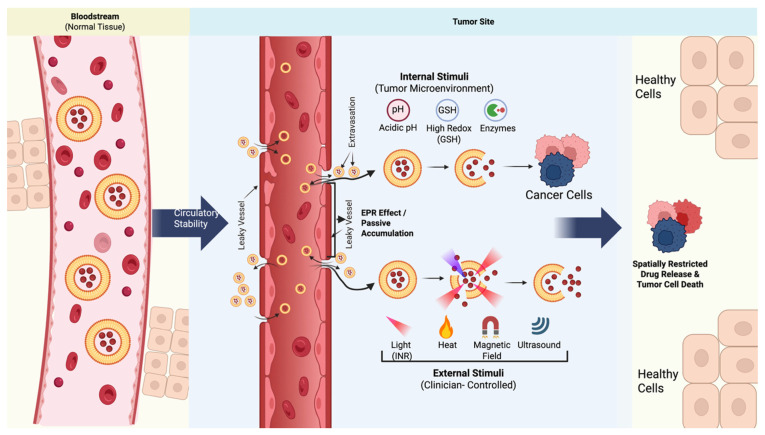
**Overview of stimuli-responsive nanocarrier-based drug delivery to solid tumors.** Drug-loaded polymeric nanocarriers are designed to maintain circulatory stability within the bloodstream of normal tissue and to selectively extravasate at the tumor site through leaky tumor vasculature (passive accumulation via the enhanced permeability and retention [EPR] effect). At the tumor site, drug release is then activated either by internal stimuli intrinsic to the tumor microenvironment such as acidic pH, elevated intracellular glutathione (GSH) levels, or overexpressed enzymes, or by clinician-controlled external stimuli, including near-infrared (NIR) light, mild hyperthermia, alternating magnetic fields, and focused ultrasound. This combination of stable systemic circulation and trigger-dependent local release achieves spatially restricted drug delivery and tumor cell death while preserving surrounding healthy cells.

The EPR effect remains one of the cornerstones of passive tumor targeting, and its role in enabling the accumulation of macromolecular medicines at tumor sites has driven the development of numerous therapeutic agents, including liposomes, polymers, micelles, and nanoparticles, ultimately leading to clinically approved treatments such as Doxil^®^ [15,16]. These agents exploit the structural abnormalities of tumor vasculature to accumulate more efficiently within the tumor mass, making EPR-mediated passive accumulation an indispensable first step in targeted delivery. It is worth emphasizing that even the most sophisticated active targeting strategies remain contingent on this initial EPR-driven accumulation—a nanocarrier must first reach the tumor before any ligand–receptor interaction can take place. Notably, smaller tumor-specific antibodies below approximately 40 kDa fall outside this framework entirely, as their rapid systemic clearance prevents meaningful tumor accumulation, further underscoring how central the EPR effect is to successful nanomedicine delivery [17,18]. At the same time, the EPR effect is far from uniform: its magnitude varies considerably across tumor types and individual patients, and is consistently lower and less predictable in human solid tumors than in the murine xenograft models on which most preclinical data are built; this translational discrepancy represents a meaningful barrier to clinical success and must be factored into the interpretation of preclinical nanomedicine efficacy data [19,20]. The overall therapeutic concept—circulatory stability, EPR-mediated tumor accumulation, and subsequent stimulus-driven drug release at the target site—is summarized in Figure 1.

### 2.2. Active Targeting via Ligand–Receptor Interactions (e.g., Folic Acid, Antibodies)

Ligand–receptor interactions are crucial for cell-to-cell communication, and their dysregulation has been linked to diseases such as cancer, autoimmune disorders, and neurodegenerative conditions [21]. Cancer cells frequently overexpress receptors that promote growth and survival, particularly those involved in oncogenic development. These receptors are useful targets for therapeutic interventions [22]. We can also utilize active techniques of targeted medication delivery, such as ligand–receptor interactions (LRIs). The use of LRIs is an active targeted drug-delivery method that precisely delivers therapeutic or diagnostic substances to a diseased location within an organ. Drug-loaded nanoparticles (NPs) designed with ligands or structural alterations selectively bind to receptors on target cells [23,24]. Ligand-mediated active targeting has revolutionized drug delivery by enabling accurate targeting at three levels: the vascular compartment (first-order), the cellular level (second-order), and the intracellular level (third-order) [25]. The target group consists of bioadhesive nonionic surfactants, antibodies, and albumin proteins [26]. This promotes selective accumulation at specific regions, thereby distinguishing pathological tissues from normal tissues [27].

Active targeting of malignant tumors can use three key cellular targets: first, cancer cells, which overexpress receptors like folate, glycoproteins, transferrin, or epidermal growth factor; second, the tumor endothelium, which upregulates integrins, vascular endothelial growth factors, adhesion molecules, and matrix metalloproteinases; and third, stromal cells, which adopt a tumor-supportive phenotype in response to cytokines within the tumor microenvironment [22].

Many recent studies have explored the use of antibodies to bind to ligands. Nanobodies, like aptamers, bind to particular cell-surface receptors, resulting in receptor-mediated endocytosis. Once internalized, the nanobody–drug conjugate (NBC) is degraded in endosomes or lysosomes, allowing the free drug to enter the cytoplasm and exert its therapeutic effect [28]. Trastuzumab emtansine (T-DM1) is an example of this; it targets HER2-positive breast cancer by attaching to HER2 receptors, entering cells via endocytosis, and releasing DM1 in lysosomes to disrupt microtubules. High DM1 levels cause apoptosis, whereas low levels hinder cell division. Resistance develops over time as a result of diminished internalization, increased recycling, poor degradation, or drug efflux, emphasizing the need for more effective treatment strategies [29].

Another example is the use of folic acid, which targets folate receptors (FRs) on tumor cells, allowing for precise medication delivery in cancer therapy. An important strategy involves linking folic acid to a hapten, which marks tumor cells as immunogenic. This method entails identifying FR-positive cells with a folate–hapten combination, which attracts anti-hapten antibodies and activates immune cells to eliminate the tumor. This technique improves immune identification in mouse models, presenting a viable option to overcome cancer’s immune evasion [28].

**Figure 2:**
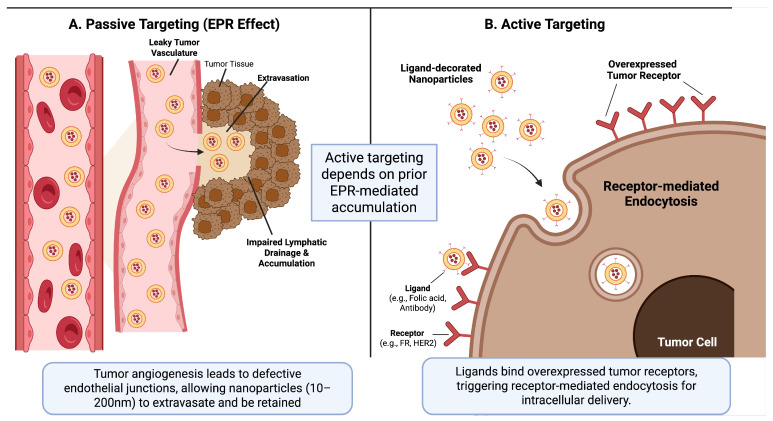
**Comparison of passive and active tumor-targeting strategies for polymeric nanocarriers.** (**A**) Passive targeting (EPR effect): Tumor angiogenesis produces structurally defective endothelial junctions in the tumor vasculature, allowing nanoparticles in the size range of approximately 10–200 nm to extravasate from leaky vessels into the tumor interstitium. Concurrent impairment of lymphatic drainage promotes their prolonged retention within the tumor mass. (**B**) Active targeting: Ligand-decorated nanoparticles (e.g., bearing folic acid or antibody ligands) selectively engage receptors that are overexpressed on the surface of tumor cells (e.g., the folate receptor [FR] or HER2), thereby triggering receptor-mediated endocytosis and intracellular delivery. Effective active targeting depends on prior EPR-mediated accumulation of nanoparticles within the tumor microenvironment.

Targeted drug-delivery systems face several challenges, including reduced targeting precision resulting from the selection of incorrect ligands, limited therapeutic efficacy even when medications reach cancer cells due to the formation of a protein corona on the delivery system surface, and adverse side effects. Furthermore, these systems are frequently complicated, inefficient, and encounter problems in clinical translation [30,31,32]. The complementary nature of passive and active targeting strategies is illustrated in Figure 2.

### 2.3. Controlled and On-Demand Drug Release Strategies (e.g., pH-Triggered Swelling, Enzyme Degradation)

Therapeutic complex formulations—such as controlled drug-delivery systems—aim to keep the medication concentration constant in the human body and in tissues for a specific amount of time [33]. These drug-delivery systems—also called smart drug-delivery systems—enable precise control over different categories such as the timing, location, and quantity of drug that is released by utilizing intrinsic or extrinsic stimuli-responsive mechanisms. These smart delivery systems can be activated by either one or a combination of triggers such as changes in pH, temperature, light exposure, specific biomarkers, electric and magnetic fields, and ultrasound. Adding to that, physical stimuli, like light, electric and magnetic fields, and ultrasound, and pharmacological stimuli, like small molecules and nucleic acids, can trigger the release of treatments from macroscale drug depots. There are specific details about these novel drug delivery techniques in [34,35].

**Figure 3:**
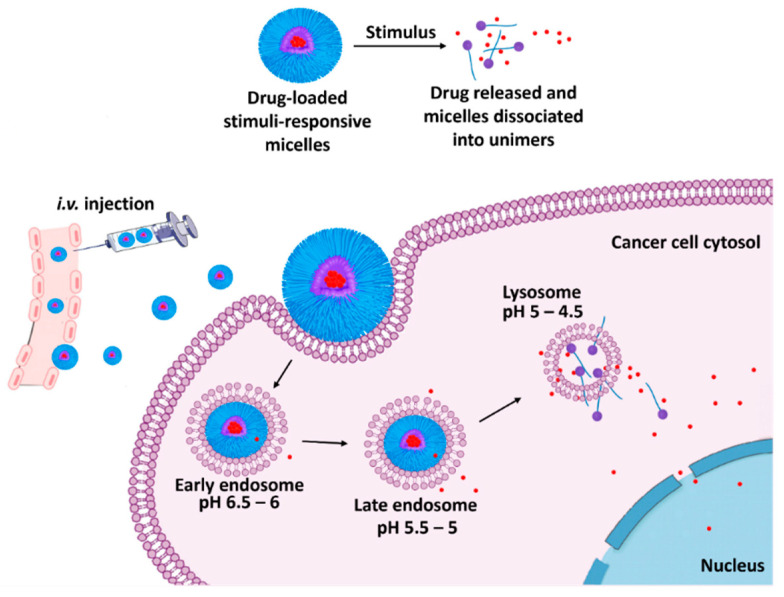
**Schematic illustration of pH-responsive drug release from polymeric micelles following cellular internalization.** After intravenous (i.v.) injection, drug-loaded stimuli-responsive micelles enter the cancer cell cytosol via endocytosis and traffic sequentially through the early endosome (pH ≈ 6.5–6), late endosome (pH ≈ 5.5–5), and lysosome (pH ≈ 5–4.5). Progressive acidification within these compartments triggers micelle dissociation into unimers, with the encapsulated drug (red dots) released into the surrounding cytosol. Maximal drug release is observed in the lysosomal compartment owing to its lower pH. Adapted from Jin et al. (2024) [37].

pH-sensitive systems are one of the best illustrations of intrinsic control. It has been demonstrated that pH-responsive micelles effectively encapsulate hydrophobic medications, allowing for an “on-demand” release at designated target regions. Micelle disintegration and the loss of ionic connections with the drug were a concern due to this method’s ionization of functional groups. Mixed-charge micelles with IEPs ranging from pH 4.65 to 9.65 have been achieved by carefully adjusting the acid and amine groups within the polymer chains, allowing us to fine-tune the micelles’ stability, surface charges, and isoelectric point (IEP). Additionally, according to diffusion-ordered spectroscopy (DOSY), the micelle size increases with temperature or as they approach their IEP. Importantly, pH variations cause the release of encapsulated doxorubicin, demonstrating the potential of these pH-responsive micelles for adaptive therapeutic delivery in applications such as cancer therapy, myocardial infarction treatment, and wound healing [36]. The progressive intracellular acidification driving this release is illustrated in Figure 3.

Another approach to the development of drug-delivery systems (DDSs) relies on thermo-responsive polymers, which respond to extrinsic stimuli for tailored on-demand treatment; in this method, heat production is utilized to induce drug release from temperature-sensitive materials. Another successful external stimulation technique is the application of mild electric fields to specified tissues following the administration of electro-responsive drug carriers, which enables precise control over on-site drug release. Electrical stimulation can control drug release through various processes, including oxidation–reduction reactions, disruption of carrier structures, and activation of thermoresponsive carriers via heat generated by electrical currents [38,39].

Research has shown the effectiveness of a two-stimulation drug release system using polypyrrole nanoparticles where the drug release is regulated by the application of electric impulses and changing the temperature. The application of external electric field at −1.5 V/cm resulted in the production of local heat and thus increased the thermal sensitivity of hydrogel and stimulated the release of drugs. Electrostimulation was observed to release about 60 ng of fluorescein every day. This approach makes accurate dosing possible and is ideal for use in the treatment of pain and cancers [40].

The market has a lot of potential for the development of innovative drug delivery systems based on responsive polymers. Although there are some startups researching the possibility of using responsive particles, their introduction into the market faces many challenges such as a lack of in vivo experiments and a lack of studies combining different stimulations. The complexity of these methods is another factor that hinders their practical applications, while ensuring their precision requires special technology [41]. Furthermore, therapies using nanofibers (NFs) encounter difficulties in getting clinical trial permission. Presently available DDSs are non-biodegradable and need to be surgically removed [41]. Furthermore, the solvents employed in electrospinning represent a risk to the NFs. Several additional problems have been identified, including higher manufacturing capacity requirements, issues guaranteeing drug–matrix compatibility, and difficulties optimizing drug release. Although NFs show promise for targeted medication release, further study is needed to help determine their commercial and clinical viability [42]. Research has proven that release systems designed to respond to multiple stimuli such as pH and redox conditions can provide better accumulation of drugs within cells compared to single-stimulus systems, although further testing is required in vivo in clinically relevant tumor models before any translation can take place [43,44,45]. Passive and active targeting approaches for tumors are summarized in Table 1.

## 3. Stimuli-Responsive Polymers in Cancer Drug Delivery

### 3.1. Internal Stimuli-Responsive Polymers (Summerized in Table 2)

#### 3.1.1. pH-Sensitive Polymers for Cancer Therapy

The first and most well-known approach involves using pH-responsive polymers that take advantage of the fact that tumor tissues cannot maintain a neutral physiological environment. Conventional drug treatment tends to fail because of widespread distribution of drugs, their toxicity to the whole body, and premature elimination from the tumor area before attaining the therapeutic concentration [46]. On the other hand, drug delivery vehicles responding to acidity will remain stable in blood plasma but decompose, swell, or undergo chain breaking in response to lower pH [47].

Most tissues function optimally at a pH range of 7.35–7.45. The pH of tumors, however, tends to drop closer to 6, a direct result of hypoxia-driven glycolysis and poor drainage [48]. Add to that the lysosomes and endosomes inside the cells, where the pH falls to 6.5–4.5, and you have multiple opportunities for selective activation [49].

There are two major strategies for designing stimuli-sensitive carriers: first is the use of acid-labile linkers such as acetal, hydrazone, benzoic imine, and ortho-ester linkages; the second strategy is the use of ionizable polyelectrolytes. In both of these approaches, the carriers are provided with the capability to respond to acidic environments, which act as a “switch” [50,51,52]. PEG-poly(aspartate)-hydrazone-adriamycin, a hydrazone-based micelle, provides an excellent example of a stimuli-sensitive system; it remains stable in the circulatory system of a body but responds to acidic conditions and is cleaved to provide a doxorubicin (DOX)-releasing system [53]. One study used poly[(D,L-lactide)-co-glycolide]-PEG with an N-Boc-histidine cap. It remained stable within the body and degraded at a pH of 6.2, which led to the specific release of DOX [54,55].

Another study described how PCCS-conjugated PEG micelles could be used to make a pH-responsive carrier with enhanced circulatory time and enhanced efficiency in delivering DOX to tumor cells [56,57].

These systems have clear promise: greater tumor drug levels with less systemic harm [58]. Still, problems remain. Manufacturing at scale, achieving consistent stability, and avoiding immune activation are all hurdles. In addition, regulators remain cautious. Some recent findings suggest that pH-responsive block copolymer micelles with engineered pKa values can achieve almost complete intracellular delivery of drugs in the endolysosome compartments, thereby proving the efficacy of this method despite the necessity of pharmacokinetic evaluation of this method [59,60]. Until then, the research moves ahead with the hope that such carriers will become part of standard treatments [61].

#### 3.1.2. Redox-Responsive Polymers

Another effective approach exploits the redox gradients that exist within tumor cells. Unlike normal cells, tumors contain glutathione (GSH) concentrations that are 100 to 1000 times higher than those outside the cell [62,63]. This disparity makes redox-responsive carriers an obvious choice as they can remain intact when circulating but break down upon entry into a cell [64].

The chemistry usually involves disulfide bonds. Built into the backbone, side chains, or drug linkers, these bonds survive in oxidative extracellular conditions [65]. However, once exposed to high GSH concentrations in the cytoplasm, they cleave, releasing the drug payload [66].

One of the strongest arguments in favor of this approach is its ability to address multidrug resistance (MDR) [61,67]. Cancer cells often upregulate efflux pumps, spitting drugs out before they can work. Redox-responsive carriers bypass this barrier by releasing drugs inside cells at concentrations high enough to overwhelm efflux [51].

There are several challenges associated with redox-responsive polymers. Premature cleavage can cause toxicity. Furthermore, inconsistency in accumulation between areas (as GSH levels can vary between tumor sites and even within regions of the same tumor) makes the accurate prediction of response and side effects difficult. Carriers must remain stable throughout the transport to the site of the tumor, yet they should only respond and be activated inside the cells of the tumor, which is a difficult balance [50,51,68]. More preclinical work is needed to ensure reliable performance at the clinical scale. Recent advances in dual pH/redox-responsive designs have shown improved spatiotemporal precision in vitro, yet robust in vivo verification of intracellular GSH-triggered release kinetics remains a prerequisite for clinical development [66,69,70].

#### 3.1.3. Enzyme-Responsive Polymers for Cancer Therapy

A third approach uses the enzymatic fingerprint of tumors. Many cancers overexpress enzymes such as matrix metalloproteinases (MMPs), cathepsins, or hyaluronidases, molecules that remodel tissue, degrade the extracellular matrix, and support invasion [71,72].

Designing polymers around this feature is conceptually simple: design carriers containing enzyme-cleavable linkers or backbones that are stable in blood but fragment in enzyme-rich environments. MMP-sensitive micelles, for example, circulate safely until they encounter tumor MMPs, which cleave peptide linkers and release DOX directly inside tumor tissues [73].

Other strategies focus on cathepsin-sensitive linkers which have been tried to deliver doxorubicin to tumor cells. Cathepsins-B-cleavable doxorubicin prodrug nanoparticles are stable nanostructures that accumulate in tumors due to the EPR effect and when endocytosed, they are selectively cleaved by lysosomal cathepsin B to release the active doxorubicin. This ensures higher intracellular concentrations while sparing healthy tissue, and shows 2.1-times greater anticancer effects than free doxorubicin [74]. Similarly, hyaluronidase-sensitive polymers composed of hyaluronic acid exploit the overexpression of CD44 on tumor cells, and uptake is through CD44 receptor-mediated endocytosis. Once internalized, abundant hyaluronidases degrade the carrier, release the drug, and simultaneously improve tissue penetration. One example is a mesoporous silica nanoparticle (MSN)-based delivery system that is responsive to hyaluronidases, which showed superiority compared to free doxorubicin in an in vitro study [75,76].

Flexibility is another advantage. Enzyme-responsive designs can be combined with pH or redox sensitivity for greater specificity [77]. The drawback is variability and intratumoral heterogeneity: enzyme levels differ not just between tumor types but also within a single tumor, reducing the uniformity of drug release [78,79,80]. Carriers must also withstand circulation long enough to reach their target. If they release early, healthy tissues take the hit [81].

**Figure 4:**
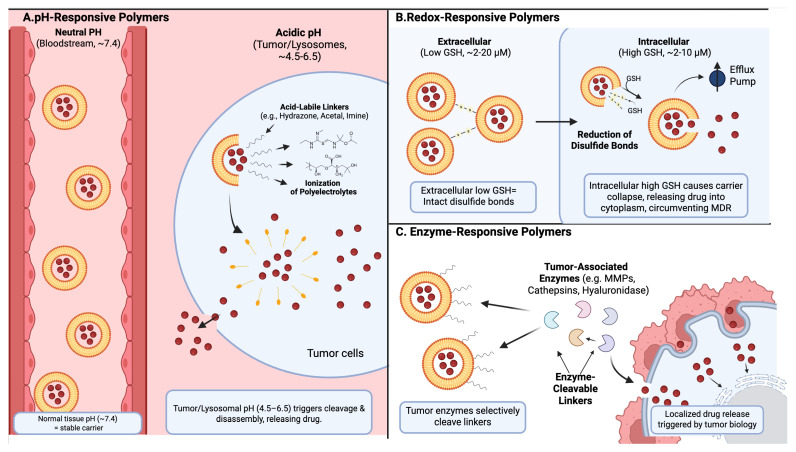
**Mechanistic basis of internal stimuli-responsive polymeric nanocarriers.** (**A**) pH-responsive polymers: Carriers remain stable at the neutral pH (≈7.4) of the bloodstream but undergo cleavage at acid-labile linkers (e.g., hydrazone, acetal, imine) and/or ionization of polyelectrolytes upon exposure to the acidic (pH ≈4.5–6.5) tumor and lysosomal microenvironment, leading to disassembly and drug release within tumor cells. (**B**) Redox-responsive polymers: The steep extracellular-to-intracellular glutathione (GSH) gradient (extracellular ≈2–20 µM vs. intracellular ≈2–10 mM) is exploited by incorporating disulfide bonds; high intracellular GSH causes carrier collapse and cytoplasmic drug release, helping circumvent multidrug-resistance (MDR) efflux pumps. (**C**) Enzyme-responsive polymers: Tumor-associated enzymes (e.g., matrix metalloproteinases [MMPs], cathepsins, hyaluronidase) selectively cleave enzyme-sensitive linkers, resulting in localized drug release that is mechanistically driven by tumor biology.

Despite these challenges, enzyme-responsive systems remain one of the most promising directions. Studies have demonstrated that MMP-2/9-cleavable peptide-conjugated polymeric micelles achieved significantly greater tumor accumulation and reduced off-target toxicity than non-responsive controls in orthotopic pancreatic tumor models [37,72]. They use the cancer’s own biology as the trigger, aligning therapy with the very pathways that tumors rely on to grow and spread. The mechanistic distinctions among pH-, redox- and enzyme-responsive systems are summarized schematically in Figure 4.

### 3.2. External Stimuli-Responsive Polymers (Summerized in Table 2)

#### 3.2.1. Temperature-Responsive Polymers

Temperature-responsive polymers occupy an interesting niche in stimuli-responsive drug delivery; a shift of just a few degrees is enough to collapse their structure and trigger release, which makes heat an appealingly simple switch. The textbook example is PNIPAM, which undergoes a phase transition at around 32 °C [82]. The catch, however, is that this transition temperature sits below normal body temperature, let alone the 40–45 °C range used in clinical hyperthermia, meaning that native PNIPAM would collapse spontaneously in the bloodstream before reaching any tumors. The practical fix is chemical modification—copolymerization or end-group engineering—to push the LCST up into the 39–42 °C window, allowing the polymer to stay soluble at 37 °C but respond to mild local heating. Done correctly, this creates a carrier that circulates quietly through the body and releases its payload only when a clinician applies heat to the tumor site, leaving the surrounding tissue untouched. More refined architectures, such as diblock copolymers with thermoresponsive segments, give researchers additional control over loading efficiency and release kinetics [82]. The clinical appeal is clear, particularly in hyperthermia-assisted regimens where controlled heating already forms part of the treatment plan. That said, the path to translation remains bumpy: off-target heating, batch-to-batch variability, and the unpredictable thermal responses seen across patients all complicate development [83]. A 2023 study offered an encouraging data point: a PNIPAM-co-acrylamide system tuned to an LCST of 41 °C stably held its payload at physiological temperature, released it rapidly under mild hyperthermia, and suppressed tumor growth by more than 80% in a murine breast cancer model [84].

#### 3.2.2. Light-Responsive Polymers

Light offers something that few other stimuli can: precise control in both time and space. In these carriers, illumination triggers photocleavable linkers or photosensitive nanoparticles, setting off drug release [85]. Some elegant systems—such as glycosylated nanoparticles—have already shown selective activity in cancer cells while leaving normal tissues untouched [86,87,88]. Ultraviolet light, although effective at cleaving bonds, has a limited penetration depth. Near-infrared (NIR) light reaches deeper into tissue and has therefore become the preferred option. Gold nanorods, upconversion nanoparticles, and semiconductor quantum dots have proven their ability to transform light into heat or reactive oxygen species, which propel drug release, thereby improving the drug’s clinical efficacy. These tactics reduce collateral damage and improve dosing accuracy [89,90]. However, practical barriers persist: light scattering in tissue and the risk of phototoxicity remain uncertain. Notably, a 2024 report described NIR-II window-responsive polymeric nano-assemblies that achieved tissue penetration depths of 1–2 cm in in vivo tissue phantoms while maintaining high spatiotemporal drug release precision [89]. Moreover, preparation of nano-formulations often involves significant amounts of time, high manufacturing costs, and inconsistent product quality, which are attributable to their very intricate and precise synthetic methodologies [89,91].

#### 3.2.3. Magnetic and Ultrasound-Responsive Polymers

**Figure 5:**
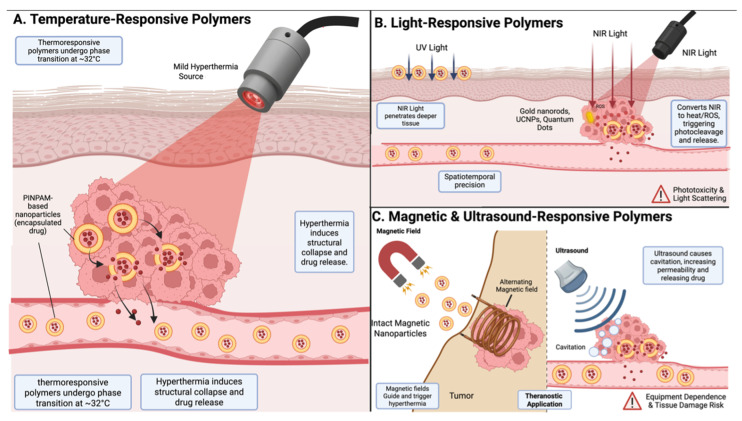
**Mechanistic basis of external stimuli-responsive polymeric nanocarriers.** (**A**) Temperature-responsive polymers: Thermoresponsive carriers (e.g., PNIPAM-based nanoparticles) undergo a phase transition at ≈32 °C; mild localized hyperthermia (≈32 °C) induces structural collapse of the carrier and triggers site-specific drug release. (**B**) Light-responsive polymers: Ultraviolet (UV) light is limited by shallow tissue penetration, whereas near-infrared (NIR) light penetrates more deeply and, when paired with photoresponsive nanomaterials such as gold nanorods, upconversion nanoparticles (UCNPs), and quantum dots, is converted into heat or reactive oxygen species (ROS) that drive photocleavage and drug release with high spatiotemporal precision; phototoxicity and light scattering remain practical limitations. (**C**) Magnetic and ultrasound-responsive polymers: Magnetic nanoparticles can be both guided to the tumor and activated by an alternating magnetic field to generate hyperthermia, while focused ultrasound induces cavitation that increases local vascular permeability and promotes drug release; both modalities support theranostic applications but depend on specialized equipment and carry a risk of off-target tissue damage.

Magnetic and ultrasound-based approaches offer non-invasive methods for targeting tumors located deep within the body. By embedding magnetic nanoparticles into polymer carriers, drugs can be guided externally and concentrated at the tumor, with alternating fields then used to heat or disrupt the carrier and release the payload [92,93]. Ultrasound achieves a similar end through a very different route: acoustic waves cause cavitation in nanogels, which increases local vascular permeability and facilitates drug penetration while also triggering release within the tumor itself [94]. In several cases, these technologies are combined with microbubbles or imaging agents, which create systems that have the ability to deliver a therapy as well as simultaneously monitor its effects. The advantages include precise targeting, real-time control, and minimal invasiveness. But as with other approaches, problems remain. Magnetic targeting requires precise field calibration, whereas high-intensity ultrasound risks collateral tissue damage. Both require specialized equipment, and consistent large-scale production is still difficult [95]. Improvements in the tumor drug concentration were observed with the use of ultrasound-sensitive polymeric nanocarriers in all the studies conducted, but the use of such methods in the clinic faces challenges due to the lack of an established exposure time for ultrasound [96,97,98]. Until this problem is solved, implementation will continue to be difficult. The principal extrinsic-trigger modalities and their representative mechanisms are summarized in Figure 5.

**Table 2:** Mechanisms and triggers of stimuli-responsive polymeric systems.

Stimulus Type	Trigger	Mechanism of Release	Examples from the Literature
**Internal (Intrinsic)**	pH (Acidity)	Polymers expand, disintegrate, or cleave acid-labile linkers (e.g., hydrazone, acetal) in reaction to the acidic tumor microenvironment (pH ≈ 6) or lysosomes (pH 4.5–6.5) [49,99].	PEG-polyaspartate formulations dissociate at acidic pH levels, releasing DOX [53]. Poly(acrylic acid) and chitosan: at acidic pH values, they undergo swelling or disintegration, releasing the encapsulated drug [99]. Translational status: mainly preclinical; currently no FDA-approved pH-sensitive polymeric nanoparticle [100,101].
	Redox (Glutathione)	When exposed to high quantities of intracellular GSH (100–1000 times higher than external levels), disulfide bonds that are part of the carrier backbone or linkers break [62,66].	Disulfide-cross-linked micelles: release DOX or paclitaxel in the cytoplasm to avoid efflux pumps [102]. Translational status: preclinical; GSH assay-based patient stratification not yet clinically validated.
	Enzymes	Tumor enzymes that are overexpressed, like MMPs, cathepsins, and hyaluronidases, break down polymer backbones or certain peptide linkers [71,72].	MMP-cleavable micelles: peptides are degraded by MMPs, releasing the payload [73]. Hyaluronidase-cleavable MSNs: degradation of these nanoparticles occurs via highly prevalent hyaluronidases post internalization through CD44 receptor [75]. Translational status: enzyme-responsive ADC (ESG401, Phase 1a) uses enzymatic cleavage; purely polymeric enzyme-responsive carriers remain preclinical [103].
**External (Extrinsic)**	Temperature	When heat is applied to thermo-responsive polymers (such as PNIPAM), they change phases at their tuned LCST (typically 39–42 °C for therapeutic use; note that native PNIPAM LCST ≈ 32 °C requires chemical modification to match clinical hyperthermia temperatures of 40–45 °C) or change their structure [82].	Thermosensitive hydrogels: they turn into a gel at 37 °C and can be used as local depots for long-term release [104]. Note: PNIPAM LCST must be tuned to 39–42 °C for clinical use; thermosensitive liposomes (LTLD) have reached clinical trials while polymeric PNIPAM systems remain preclinical [105].
	Light	Photocleavable linkers or photosensitizers are activated by UV or NIR light, which creates heat or ROS that breaks the carrier [85,89].	Gold nanorods/upconversion NPs: convert light energy to heat/ROS to trigger release [89]. Clinical translatability: PHOTOFRIN^®^ PDT is a clinically approved treatment modality (not using polymer-based NCs)[106]; NIR-responsive polymeric NCs are still at preclinical stage. Depth of tissue penetration: UV < 1 mm; NIR ≈ 1–2 cm [89].
	Magnetic/Ultrasound	External fields guide magnetic carriers or create sonic cavitation/hyperthermia to initiate release [92,93,94].	Magnetic nanoparticles: driven by external fields and heat-activated for drug release [107]. Antibubbles/Nanogels: subjected to ultrasound to cause cavitation or leakage [108]. Clinical translation: magnetic hyperthermia using iron oxide nanoparticles (NanoTherm^®^, sourced from MagForce AG, Berlin, Germany) is approved in Europe for glioblastomas [109], but polymeric ultrasound-responding carriers are still pre-clinical. Both require specialized equipment.

**Abbreviations:** pH, potential of hydrogen; DOX, doxorubicin; GSH, glutathione; MMP, matrix metalloproteinase; MSNs, mesoporous silica nanoparticles; PNIPAM, poly(N-isopropylacrylamide); UV, ultraviolet; NIR, near-infrared; ROS, reactive oxygen species; NPs, nanoparticles.

### 3.3. Types and Uses in Cancer Treatment

There are many different types of stimuli-responsive polymers, which can be grouped based on the type of trigger they respond to. Some depend on the tumor itself, which is what we refer to as “internal stimuli.” Some polymers need an outside force or energy that the physician controls. This tiny difference makes a very broad field clearer.

Internal systems use tumor biology directly. For instance, pH-responsive carriers capitalize on the acidic environment that many solid tumors exhibit. Poly(acrylic acid) and chitosan-based structures are classic designs that swell or break down when the pH decreases, releasing their drug payload only when the acidity is high [99]. Redox-responsive systems operate on a distinct principle: they exploit the elevated intracellular glutathione (GSH) levels present in cancer cells. Adding disulfide bonds to the carrier helps these polymers remain together while in the blood but break apart within the tumor, allowing for tightly controlled release [110]. A third approach centers on enzymes. Proteases, such as matrix metalloproteinases (MMPs), are often overexpressed in tumors. These enzymes break down polymer backbones or cleavable linkers, which allows medications to be released exactly where they are needed in tumor tissue [92].

External triggers add another level of control, allowing the doctor treating the patient to directly regulate when and how a medicine is activated. For instance, temperature-responsive polymers are designed to release their contents when the tissue is slightly heated. This is typically done with hyperthermia therapies [83]. Light-responsive methods offer enhanced spatial resolution, utilizing ultraviolet or near-infrared light to trigger the release of medication in a precisely defined area [111]. Some carriers have magnetic nanoparticles or parts that are sensitive to ultrasound. In these systems, an external magnetic field or focused ultrasound may both direct the carrier and discharge the payload. This makes it possible to target deep tissue without having to do intrusive treatments [94].

These various methods are useful not just because they are new, but also because they can be applied in various ways. Internal triggers connect therapy to tumor biology, whereas external triggers provide you with accuracy when you need it. Nanocarriers and hydrogels are increasingly combining both techniques, resulting in systems that are more adaptable and clinically significant. Some of these formulations are no longer limited to the laboratory; they have progressed to preclinical testing and early-stage clinical trials [95].

## 4. Clinical Studies of Stimuli-Responsive Polymers in Cancer Therapy

Using tumor-specific triggers such as pH, enzymes, redox conditions, temperature, and light has given stimuli-responsive polymers the opportunity to become a new game-changer in cancer therapy. These systems represent promising developments in precision medicine and treatment for a variety of cancer types through increasing treatment efficacy while at the same time minimizing off-target effects [112].

### 4.1. pH-Responsive Polymers

Recent studies have highlighted the potential of pH-sensitive polymers in cancer therapy, demonstrating their ability to enhance treatment outcomes while minimizing adverse effects. These tailored delivery methods selectively release medicines in tumor microenvironments, with promising in vivo outcomes. One strategy for glioblastoma treatment is a pH-responsive nanoplatform that combines lactoferrin, titanocene, and a ZIF-8 metal–organic framework (ZIF-8@Lf-TC), supplemented with a lenalidomide-HA conjugate. Characterization validated its stability, and in vitro experiments revealed higher cytotoxicity in U87MG and RAW264.7 cells, as well as increased reactive oxygen species (ROS) levels and disruption of inflammatory cytokines. This dual-drug approach has shown encouraging results in reducing cancer growth [113].

A further experiment showed the application of the pH-sensitive metal–organic framework nanoparticle approach in in vivo cancer immunotherapy with B16-OVA melanoma cells. The outcome of this therapy led to approximately 100% survival in tumor-bearing mice through the stimulation of CD8+ cytotoxic T-lymphocyte activity and improved immune cell recruitment. No significant toxicity was reported in the experiment, showing that this approach can be applied safely in the treatment of cancer [114].

Finally, researchers investigated pullulan–adipodihydrazide–doxorubicin (Pu-DOX) nanoparticles for the targeted administration of pyrrolidinedithiocarbamate (PDTC) and doxorubicin (DOX) in hepatocellular carcinoma. These nanoparticles are stable and release drugs rapidly in acidic conditions, efficiently triggering apoptosis in both DOX-sensitive and resistant HepG2 cells and reducing tumor development in vivo. This dual-drug delivery technology has tremendous potential for overcoming chemoresistance and improving treatment success. Together, these new technologies represent significant advances in targeted cancer therapy, opening the door to more effective and safer treatment alternatives [115].

### 4.2. Enzyme-Responsive Polymers

In a phase Ia study, ESG401, an ADC comprising a trop-2-targeting antibody coupled with SN-38, was investigated in 40 patients suffering from advanced metastatic solid tumors, mainly breast cancers. The highest tolerable dose was found to be 16 mg/kg on days 1, 8, and 15 within a cycle of 28 days. In 38 patients who were evaluable for efficacy assessment, the ORR was 34.2%, DCR was 65.8%, and CBR was 50% (SD ≥ 6 months). Clinically feasible doses of ESG401 increased the ORR to 40.6%, DCR to 75%, and CBR to 56.3%. This study demonstrated a favorable safety and clinical response to the drug [103].

Another study describes a unique strategy for targeted medication delivery for pancreatic cancer using the protease MMP-9 via a polymeric nanocarrier (Magh@PNPs-PEG-RegaCP-PEG) with an MMP-9-cleavable linker. Both in vitro and in vivo tests demonstrated that this carrier’s absorption is largely dependent on MMP-9, with around 80% accumulation in pancreatic tumors and reduced liver metastasis. These findings highlight the potential of MMP-9 to enhance the selectivity and efficacy of cancer-targeted treatments [116].

### 4.3. Redox-Responsive Polymers

A study found that PAA-ETB-NPs, a redox-responsive, pH-sensitive nanoparticle technology, enhanced erlotinib (ETB) administration for NSCLC treatment in vivo. PAA-ETB-NPs (170 nm, −32 mV) demonstrated higher encapsulation efficiency (>85%) and increased cytotoxicity compared to ETB-NPs and free ETB. In tumor-bearing animals, PAA-ETB-NPs significantly enhanced tumor suppression without causing any harm. This nanoparticle approach enhances ETB’s stability and effectiveness, making it a promising lung cancer therapy [117]. Another work demonstrated the production of redox-responsive poly(allylamine)/Eudragit S-100 nanoparticles (PAEU NPs) via air oxidation, without the use of external cross-linking agents. PAEU NPs showed outstanding encapsulation efficiencies of around 85% for doxorubicin and 97% for curcumin. The experimental results showed that these nanosystems killed HCT116 cancer cells more efficiently than the individual medicines. In vivo biodistribution experiments in Balb/C mice demonstrated that PAEU NPs efficiently targeted the colon, highlighting their potential as a therapeutic approach for colorectal cancer [118].

### 4.4. Temperature-Responsive Polymers

According to a study, intratumoral delivery of a dual-drug-loaded thermosensitive hydrogel (NCTD-NPs + DOX) improved HCC therapy. In H22 tumor-bearing mice (*n* = 6 per group), the hydrogel decreased tumor development by 72%, reduced side effects, and enhanced survival by 40% over free medicines. Reduced tumor growth and angiogenesis were indicated by 55% and 60% lower (*p* < 0.05) levels of Ki-67 and CD31 expression, respectively. The hydrogel remained liquid at 25 °C until gelling at 37 °C, enabling long-term drug release and making it a viable local HCC treatment [104].

For unresectable hepatocellular carcinoma (HCC) tumors (3–7 cm), the study compared radiofrequency ablation (RFA) with lyso-thermosensitive liposomal doxorubicin (LTLD). Out of 22 patients, those on LTLD had a higher ablation success rate (93.3%) and a larger coagulation volume (105.7 cm^3^ vs. 37.3 cm^3^). The mean overall survival (OS) was also higher at 68.5 months compared to 46.0 months with RFA alone. Despite no added harm from LTLD, four patients withdrew from the research, indicating retention issues. These findings suggest that LTLD improves outcomes; however, further optimization is required for wider adoption [119].

### 4.5. Light-Responsive Polymers

Twelve elderly patients with aggressive, recurrent non-melanomatous skin cancers were treated with photodynamic therapy (PDT) mediated by PHOTOFRIN. By injecting 1.0 mg/kg of PHOTOFRIN and activating it with laser light, PDT selectively targets and eliminates tumor cells while preserving healthy tissue. Of the twelve patients treated, ten had complete responses, demonstrating great wound-healing and cosmetic outcomes. Given its non-invasive nature and effectiveness, particularly in patients who have already had major surgeries and radiation, PDT appears to be a viable locoregional option for challenging skin malignancies [120].

Another study developed BUC@ZMS, a tumor-targeted core–shell nanoparticle coated with BxPC-3 cancer cell membranes, which combines photodynamic therapy (PDT) with chemodynamic therapy (CDT) for pancreatic ductal adenocarcinoma (PDAC). The nanoparticles, composed of upconversion nanoparticles and Zn1–xMn(x)S, produced reactive oxygen species (ROS) under near-infrared (NIR) light stimulation, depleted glutathione (GSH), and increased oxidative stress. In vivo, BUC@ZMS demonstrated homologous tumor targeting, with a blood circulation time of 3.51 h and preferential tumor accumulation. Under 1 W/cm^2^ NIR light, tumor growth was reduced by ≈90% with no damage. This dual-therapy method has strong potential for treating PDAC by utilizing photosensitive nanoparticles for targeted and efficient therapy [121].

### 4.6. Magnetic and Ultrasound-Responsive Polymers

Twenty-four studies that were published up until November 2022 were examined in a comprehensive review of magnetic nanoparticle-based hyperthermia (MNH) for cancer treatment. MNH treats cancers, including pancreatic, breast, and liver cancers, by using nanoparticles like iron oxide (Fe_2_O_3_, Fe_3_O_4_)- and liposome-conjugated nanoparticles. The studies’ findings demonstrate a 30% improvement in life expectancy for pancreatic cancer and a 33% increase in tumor-ablation rates for other malignancies. Although encouraging, more research is required to increase MNH’s applicability and boost its effectiveness as an additional cancer treatment [107].

In vivo investigations have demonstrated that antibubble-loaded macrophages facilitate targeted, ultrasound-triggered drug release. When CreERT2xGLOVA reporter mice were subjected to diagnostic ultrasonography (21 MHz), bioluminescence revealed that 4-hydroxytamoxifen (4-OHT) was released locally. Antibubbles remained stable for 48 h, with no spontaneous leakage, and ultrasound caused accurate load release at the injection site. This technique combines macrophage migration to inflamed tissues with spatiotemporal control via ultrasound, resulting in a potential strategy for targeted drug administration with minimal off-target effects [108].

Another study investigated the use of bubble liposomes coupled with ultrasound for IL-12 gene delivery in cancer treatment. In vivo, this method increased local IL-12 expression, resulting in a dramatic reduction in tumor development in OV-HM tumor-bearing mice. Repetitive therapy resulted in approximately 80% tumor reduction, which was mediated by CD8+ T lymphocytes, as evidenced by depletion studies. Ultrasound-triggered bubble-liposome collapse increased gene-delivery effectiveness, indicating a viable non-viral vector approach for targeted, immune-mediated cancer treatment with low systemic adverse effects [122].

Despite the potential of “smart” polymer systems in tissue engineering, drug delivery, and diagnostics, cellular toxicity, the accumulation of non-biodegradable polymers (like PNIPAAm), and high clinical trial costs are obstacles to clinical translation. Investment is limited by the need for FDA-approved polymers (like PEG and PLGA) [119,123]. In addition to biological barriers like opsonization, MPS clearance, and endosomal escape, toxicity, limited absorption, and clinical translation continue to be challenges for stimuli-responsive nanocarriers [1,124].

Constraints in the production process (such as reproducibility, scalability), along with biosafety considerations, limit the potential implementation of this method, hence the need for an effective regulatory framework [125]. The area of stimulus-responsive drug delivery vehicles is still under development and needs to be studied further in order to overcome some barriers.

## 5. Limitations, Future Perspectives, and Additional Challenges

Because the preceding sections are organized by stimulus modality, the relative translational maturity of these systems is not always apparent from the mechanistic discussion alone. To make this explicit, the systems are collated in Table 3 based on four translational dimensions rather than by trigger type: release validation, implementation status, patent maturity, and comparative translational readiness.

**Release-validation hierarchy.** The evidence for triggered release can be graded across four ascending levels, each more demanding than the last: (i) measurement of release in vitro; (ii) confirmation that the stimulus itself drives release within cells; (iii) demonstration in animals that the carrier reaches the tumor and reduces its growth; and (iv) verification in patients that release is genuinely trigger-dependent. Almost all the carriers, especially the polymeric carriers, fall within levels (i)–(iii), and none have reached the fourth stage. Consequently, the data constitute indirect evidence and pre-human trials rather than confirmation that these systems perform as intended in humans.

**Implementation status.** The only systems reported in Table 3 that have reached clinical use (ESG401 (antibody drug conjugate), LTLD (thermosensitive liposome), PHOTOFRIN (small-molecule photosensitizer), and iron-oxide magnetic-hyperthermia systems) are nonpolymeric comparators included for context. All the stimuli-responsive nanocarriers based solely on polymeric systems shown in Table 3 remain preclinical candidates, indicating a significant disparity between mechanistic proof of concept and clinical translation.

**Comparative translational readiness.** In terms of an integrated approach, it is apparent that internal stimulus classes (pH, redox, and enzyme) are conceptually mature but require clinical evidence, while external stimulus classes (heat, light, magnetism, and ultrasonic energy) find application for the most part through nonpolymeric drug delivery systems that are used with existing hardware. On this comparative reading, a very limited number of polymeric stimuli-responsive classes currently exceeds low-to-moderate translational readiness, and the principal obstacles to advancement are validation, reproducibility, and manufacturing rather than mechanistic novelty.

In the pharmaceutical and oncology sectors, there is a focus on the development of external stimuli-responsive polymers, as they are showing promising results in targeted therapy. This is because of their many advantages, including enabling clinicians to couple drug release with highly localized triggers and tailoring treatment to the unique characteristics of tumor microenvironments [1]. Still, the application in humans and deployment in markets are not straightforward. Biocompatibility and biodegradability remain central concerns. Unless degradation products are safe and efficiently cleared, long-term use will be limited [126]. Considerable effort is therefore being devoted to modifying polymer architectures and surface chemistry to improve their compatibility with living systems [Table 4].

Another critical issue is precision. A carrier must respond predictably to a trigger while avoiding activation in normal tissue. Achieving this balance requires advances in polymer chemistry because of its complexity; their structural complexity and the challenges associated with large-scale synthesis may slow or even limit successful translation from experimental studies into routine clinical use [50]. Failure here risks undermining the very rationale for these platforms [Table 4].

Regulation and commercialization pose an entirely different set of obstacles. Moving from bench to bedside means navigating lengthy preclinical and clinical studies that can convincingly establish safety, efficacy, and reproducibility [125]. Agencies demand standardized protocols, yet stimuli-responsive systems are diverse and often hybrid in nature. Material scientists, biomedical engineers, and regulatory experts must collaborate closely if approval pathways are to keep pace with the evolving needs of the field. Manufacturing adds further weight: cost-effective scale-up without compromising intricate polymer design remains a major bottleneck [127] [Table 4].

The challenges are real, but so are the opportunities that are beginning to take shape. Pairing stimuli-responsive polymers with immunotherapy is one direction generating genuine excitement; the two approaches appear to reinforce each other, with smarter drug delivery amplifying immune activation and the immune response improving therapeutic outcomes [128]. Drug resistance, which has long been one of chemotherapy’s most stubborn problems, may also yield to multi-mechanism designs that simultaneously hit cancer cells through more than one pathway. A 2024 analysis confirmed that pH/redox dual-responsive systems are the furthest along clinically among multi-stimuli nanocarriers, with several formulations already in Phase I trials, though regulatory approval remains elusive for all of them [61]. The next generation of polymer architectures with carriers engineered to sense and respond to two or more triggers at once could enable the precision and the adaptability that clinical oncology has been waiting for [129,130].

The picture that emerges is mixed but hopeful. The scientific foundation is strong and the potential impact is considerable, yet the path to clinical adoption will require careful resolution of technical, regulatory, and commercial challenges. These polymers cannot progress from experimental novelty to truly life-saving therapies unless these are addressed simultaneously.

## 6. Conclusions

Beyond proof-of-concept studies, stimuli-responsive polymers have been explored across numerous experimental cancer treatments; to date, however, these applications remain almost entirely preclinical rather than established therapies. Through association with tumor-specific stimuli, drugs can be delivered in a more controlled and predominantly demand-based manner. These stimuli include inherent factors such as pH, redox, and enzymes, as well as external triggers such as heat, light, magnetic energy, and ultrasonic waves. With both classes of stimuli, preclinical studies have suggested the potential for higher delivery precision and reduced systemic exposure by synchronizing drug release with disease pathophysiology or applied external energy. These advantages, however, have thus far been predominantly demonstrated in animal models and have not yet been confirmed in controlled clinical trials.

pH- and enzyme-responsive systems are particularly popular because they capitalize on characteristics that are nearly universal in the tumor microenvironment, whereas externally stimulated carriers add a layer of spatiotemporal precision and, in some situations, naturally integrate with imaging or ablative platforms. Nonetheless, translation remains inconsistent because of tumor-to-tumor variability in the EPR effect, interference with protein-corona formation, and endosomal sequestration, which will continue to limit effective bioavailability. Slow-degrading polymers often exhibit repeatability issues and an increase in safety issues due to the scale-up needed for clinical production. Hybrid, multi-stimuli designs blur the usual line between a medication and a device, creating a need for new assessment frameworks and goals that are therapeutically valid. Regulators face a dilemma as a result.

A new practical roadmap is being developed. For instance, the biodegradable backbone formulations based on PEG or PLGA materials with known kinetics under physiological conditions and incorporation of endosomolytic groups could enhance cytoplasmic delivery and should be priorities in the near future. In order to minimize variation in the delivery process and improve closed loop activation, carriers could be coupled with companion diagnostics through measurements of physiological parameters such as pH, reduction potential, or enzyme presence. Additionally, pharmacodynamic markers could be used to ensure successful activation in vivo. Clinical trials need to move away from an all-inclusive design toward phenotype-related indications based on mechanisms (e.g., cancers with high MMP or GSH contents). Finally, production of reproducible particle properties on a large scale would benefit from continuous production coupled with strict quality assurance/quality control procedures.

In theory, by using stimuli-responsive polymers in a cooperative way, it will be possible for oncology treatment to move towards an effect-based paradigm instead of an exposure-based approach; such potential does not yet constitute a clinical reality. The reasonable thing to expect in the near future is a second watershed point following the synergy of developments in the field of materials science, diagnostic techniques, and regulatory measures, rather than a revolutionary development. Considering the number of challenges stated above—particularly, the limited number of clinically approved stimuli-responsive drug delivery systems, the minimal human validation of release profiles, inadequate intellectual property protections and production processes, and the concerns about reproducibility and safety—it would be unwise to classify these platforms as clinically proven.

## Figures and Tables

**Table 1:** Classification of tumor-targeting strategies.

Targeting Strategy	Mechanism of Action	Key Characteristics and Requirements	Limitations
**Passive Targeting**	**Enhanced Permeability and Retention (EPR) Effect**	It depends on angiogenesis-induced defects in the endothelium and the higher vascular permeability in solid tumors [9,10]. Macromolecules are kept in the body because lymphatic drainage is blocked, which makes them more available at the tumor site [11]. The best particle size is between 10 and 200 nm to keep the liver and RES from quickly clearing them out and to make sure they stay in the kidneys (>6 nm) [12].	Efficacy differs because tumors are different from each other and depend on the unique tumor microenvironment. EPR does not help smaller antibodies (<40 kDa), and they are quickly removed from circulation [17,18]. Initial accumulation through EPR is necessary for later active targeting [15,16].
**Active Targeting**	**Ligand–Receptor Interactions (LRIs)**	Utilize ligands (e.g., folic acid, antibodies, aptamers) to bind receptors that are overexpressed on cancer cells [23,24]. Affects the vascular compartment, and at the cellular level and the intracellular level [25]. Facilitates receptor-mediated endocytosis, enabling NBCs to break down in lysosomes and liberate free drug [28].	The creation of a “protein corona” on the surface of the nanocarrier can hide ligands and make targeting less accurate [30]. Resistance may arise through diminished internalization, enhanced recycling, inadequate degradation, or drug efflux [29].

**Abbreviations:** EPR, enhanced permeability and retention; RES, reticuloendothelial system; kDa, kilodalton; LRIs, ligand–receptor interactions; NBC, nanobody–drug conjugate.

**Table 3:** Summary of clinical and preclinical studies of stimuli-responsive polymeric systems in cancer therapy.

Therapeutic System	Stimulus	Indication (Model)	Evidence Level	Clinical Status	Commercial Implementation	Key Findings and Efficacy Outcomes
**ZIF-8@Lf-TC + Lenalidomide-HA** [113]	pH	Glioblastoma (U87MG cells)	(i) In vitro	Preclinical (in vitro only)	None (research stage)	Elevated reactive oxygen species levels and perturbation of inflammatory cytokines. Analysis validated stability and significant cytotoxicity in vitro.
**MOF-OVA@CpG** [114]	pH	Melanoma (B16-OVA)	(ii) Preclinical (murine model)	Preclinical (murine)	None (research stage)	Demonstrated approximately 100% survival in tumor-bearing mice. Enhanced activation of CD8+ cytotoxic T cells and immunological recruitment without significant damage.
**Pu-DOX (Pullulan-DOX) + PDTC** [115]	pH	Hepatocellular Carcinoma (HepG2)	(i)/(ii) In vitro + preclinical (in vivo xenograft)	Preclinical (in vivo xenograft)	None (research stage)	Rapid release in acidic environments induced apoptosis in both chemotherapy-sensitive and resistant cells. Overcame chemoresistance and diminished tumor progression in vivo.
**ESG401 (Trop-2 ADC)** [103]	Enzyme	Metastatic Solid Tumors (Breast)	(iii) Clinical trial (Phase 1a)	Phase 1a (advanced solid tumors)	Not marketed; non-polymeric ADC	ORR of 40.6% and DCR of 75.0% at clinically significant doses; system exhibited a favorable safety profile. Note: ESG401 is an antibody–drug conjugate (ADC), not a polymeric stimuli-responsive nanocarrier (included for translational context).
**Magh@PNPs (MMP-9 linker)** [116]	Enzyme	Pancreatic Cancer	(ii) Preclinical (in vivo)	Preclinical (in vivo)	None (research stage)	≈80% of accumulation in tumor was dependent on MMP-9 presence. Reduced liver metastasis in vivo.
**PAA-ETB-NPs** [117]	Redox/pH	NSCLC (Lung Cancer)	(ii) Preclinical (tumor-bearing animals)	Preclinical (in vivo)	None (research stage)	Encapsulation efficiency exceeded 85% with significantly enhanced tumor suppression compared to free medication.
**PAEU NPs** [118]	Redox	Colorectal Cancer	(i)/(ii) In vitro + preclinical (Balb/C mice)	Preclinical (in vivo)	None (research stage)	Effectively targeted the colon in Balb/C mice. Exhibited superior efficacy in eliminating HCT116 cells compared to single pharmaceuticals.
**NCTD-NPs + DOX hydrogel** [104]	Temp	Hepatocellular Carcinoma (H22)	(ii) Preclinical (H22 tumor-bearing mice)	Preclinical (in vivo)	None (research stage)	Decreased tumor proliferation by 72% and extended survival by 40% compared to unencapsulated medications. Angiogenesis reduced, with CD31 expression decreased by 60%.
**LTLD (Lyso-thermosensitive Liposomal DOX)** [119]	Temp	Unresectable HCC	(iii) Clinical trial (randomized controlled trial, n = 22)	Clinical trials; class reached Phase III	Not marketed; non-polymeric (liposome)	Enhanced ablation success rate (93.3%) and increased coagulation volume compared to RFA alone. Mean overall survival increased to 68.5 months vs. 46.0 months for RFA. Note: LTLD is a thermosensitive liposomal formulation, not a polymeric stimuli-responsive nanocarrier (included for translational context).
**PHOTOFRIN (PDT)** [120]	Light	Non-Melanomatous Skin Cancer	(iii) Clinical (retrospective case series, n = 12)	Approved/clinically used (porfimer sodium)	Marketed; non-polymeric photosensitizer	10 of 12 elderly individuals attained a full response. Excellent wound healing and esthetic results. Note: PHOTOFRIN-PDT is a small-molecule photosensitizer, not a polymeric stimuli-responsive nanocarrier (included for translational context).
**BUC@ZMS (core–shell NP)** [121]	Light (NIR)	PDAC (Pancreatic)	(ii) Preclinical (in vivo murine model)	Preclinical (in vivo)	None (research stage)	Tumor growth decreased by approximately 90% with 1 W/cm^2^ near-infrared irradiation. Produced reactive oxygen species and reduced glutathione levels.
**Bubble Liposomes + IL-12 Gene** [122]	Ultrasound	OV-HM Tumor-Bearing Mice	(ii) Preclinical (OV-HM tumor-bearing mice)	Preclinical (in vivo)	None (research stage)	Approximately 80% tumor shrinkage facilitated by CD8+ T cells. Ultrasound-induced bubble collapse augmented gene delivery.
**Magnetic Nanoparticle Hyperthermia (MNH) (systematic review)** [107]	Magnetic	Pancreatic, Breast, Liver Cancers	Systematic review of 24 preclinical and clinical studies	Clinically applied (magnetic hyperthermia)	NanoTherm^®^ approved in EU (glioblastoma); polymeric carriers none	30% improvement in life expectancy for pancreatic cancer; 33% enhancement in tumor-ablation rates for additional cancers.

**Abbreviations:** ROS, reactive oxygen species; CD8+, cluster of differentiation 8 (cytotoxic T cells); Pu-DOX, pullulan-adipodihydrazide-doxorubicin; PDTC, pyrrolidinedithiocarbamate; ADC, antibody–drug conjugate; ORR, objective response rate; DCR, disease control rate; NSCLC, non-small cell lung cancer; PAEU, poly(allylamine)/Eudragit S-100; NCTD, norcantharidin; HCC, hepatocellular carcinoma; LTLD, lyso-thermosensitive liposomal doxorubicin; RFA, radiofrequency ablation; PDT, photodynamic therapy; PDAC, pancreatic ductal adenocarcinoma; MNH, magnetic nanoparticle hyperthermia. Note: Entries are categorized by evidence level: (i) in vitro, (ii) preclinical/in vivo animal study, (iii) clinical trial. Non-polymeric or non-stimuli-responsive systems (e.g., PHOTOFRIN PDT, LTLD liposomes) are included for translational context but do not represent polymeric stimuli-responsive nanocarriers per se.

**Table 4:** Major challenges to clinical translation of stimuli-responsive polymeric nanocarriers.

Challenge Category	Specific Barriers	Impact on Development	Proposed Solution
**Biological/Physiological**	**Tumor heterogeneity**	The heterogeneity of pH, enzymatic activity, and glutathione concentration in the same tumor as well as between different tumors presents a barrier to the even delivery of drugs [51,61].	Implement companion diagnostics to differentiate between different microenvironment phenotypes in tumors.
	**Clearance and toxicity**	Rapid elimination by the reticuloendothelial system (RES) and potential toxicity owing to premature drug release and non-biodegradable polymer degradation products [1,126] affects drug delivery.	The use of PEGylation, zwitterionic surfaces, and biodegradable PLGA/PEG scaffolds approved by the FDA.
	**Cellular barriers**	Challenges related to opsonization, endosomal escape, and protein corona formation restrict targeting accuracy; protein corona formation can mask targeting ligands and reduce cellular uptake [1,30,124].	Surface modification and endosomal escape strategies can reduce protein corona formation and improve cellular uptake and targeting efficiency.
**Manufacturing/Technical**	**Scalability and reproducibility**	Challenges in achieving uniform large-scale manufacturing of intricate hybrid polymer systems [50,95].	Continuous manufacturing platforms with real-time PAT (process analytical technology); microfluidic scale-up for nanoparticle production.
	**Stability**	These systems need to be stable during circulation and be highly sensitive at their targets, a challenging balance to reach.	Stimuli-responsive PEG detachment systems and lyophilization for long-term stability [7,50,51,54]
**Regulatory/Commercial**	**Regulatory frameworks**	The absence of established protocols and evolving frameworks for hybrid “drug-device” combinations generate regulatory ambiguity.	Early FDA/EMA pre-submission meetings; utilize 505(b)(2) pathway for nano-formulations of approved drugs [7,50].
	**Clinical trial costs**	High expenses and the need for complete safety and efficacy information limit investments [125].	Basket study design stratified by molecular characteristics (e.g., MMP high, GSH high tumors) to lower sample sizes; take advantage of patient selection based on biomarkers.

**Abbreviations**: GSH, glutathione; RES, reticuloendothelial system.

## Data Availability

No new data were created or analyzed in this study. Data sharing is not applicable to this article.
